# A Non-Exercise Model for Predicting Cardiovascular Risks among Apparently Healthy Male Office Workers—Cross-Sectional Analysis: A Pilot Study

**DOI:** 10.3390/ijerph19052643

**Published:** 2022-02-24

**Authors:** Emilian Zadarko, Maria Zadarko-Domaradzka, Zbigniew Barabasz, Marek Sobolewski

**Affiliations:** 1 Institute of Physical Culture Sciences, Medical College of Rzeszów University, University of Rzeszów, 35-959 Rzeszów, Poland; mzadarko@ur.edu.pl (M.Z.-D.); zbarabasz@ur.edu.pl (Z.B.); 2 Department of Quantitative Methods, Rzeszów University of Technology, 35-959 Rzeszów, Poland; msobolew@prz.edu.pl

**Keywords:** disease prevention, cardiovascular diseases, CRF, risk prediction, FIT Treadmill Score

## Abstract

The health condition of working-age males in Poland remains largely associated with long-lasting sick leaves, one of the main reasons of which being cardiovascular diseases (CVD). The aim of this work was to develop a prediction model for FIT Treadmill Score (“FIT” refers to Henry Ford ExercIse Testing (FIT) Project) that only depends on easily accessible somatic data and smoking without the need to perform the exercise test anymore. The study comprised 146 men with a negative cardiological history, aged 26–60, with desk-jobs. By means of regression analysis it was tested to what degree obesity-related indices as well as smoking cigarettes allow for determining the measure level of mortality risk, without the necessity of performing an exercise test. The following independent variables were entered into the linear regression model: age, BMI, Fat%, waist circumference (WC), waist to height ratio (WHtR) as well as smoking. Statistically significant factors were singled out from among them. The obtained model accounts for a significant part (over 87%) of the variability of the mortality risk measure among the tested population. Based on the value of the standardised regression coefficient *β*, it can be stated that age is the factor that mostly determines the mortality risk measure, followed by the WHtR and smoking. The simplicity of the worked-out model and, resulting from it, the possibility of its common application should enable better health monitoring of working-age men with regard to cardiovascular disease occurrence and, related to it, mortality risk, thereby improving the quality of public health management.

## 1. Introduction

High mortality of working-age people constitutes a serious problem of the Polish population, both demographically and economically [1]. The mortality risk of Polish males aged 30–59 is approximately two-thirds higher than the EU average [2]. Cardiovascular diseases, constituting the main life and health hazard of the Polish population, are considered one of the most important health problems in Poland, especially in the case of working-age males [3]. Less than half of the employers in Poland declare their concern for their employees’ health to a degree exceeding that required by the labour law. That concern is mostly expressed through their actions regarding physical working conditions and providing medical healthcare as well as—to a lesser degree—promotion of physical activity [4]. The results of many studies indicate a positive influence of physical activity, mostly leisure time physical activity, on decreasing the number of sick leaves among the employees [5]. The results of intervention programmes promoting physical activity in a workplace vary—in most cases their impact is positive, in particular in the scope of musculoskeletal complaints, improvement of physical fitness and body mass control [6]. Despite the employers’ growing awareness of economic benefits of promoting health among the employees in Poland, a serious hindrance still remains in the form of non-adjustment of health promoting programmes to the objective health needs and little interest of the employees in health [7].

Conscious actions and engagement of the employee towards the prevention of involutionary processes combined with the employer’s policy in that respect may effectively lead to a change of lifestyle into a healthier one and to the improvement of work efficiency [8].

It follows from a report by the Institute of Occupational Medicine in Poland and by the National Centre for Workplace Health Promotion concerning the employees of medium-sized and big companies in Poland that only every second employee has a proper body mass, with 39% being overweight and 9% obese. Excessive body mass is a predominantly male feature. The overweight and obese workers often sit a lot, and rest lying, at the same time more rarely exercising intensively for more than 1 h a week [9]. Desk-job workers are identified as a high-risk group for chronic diseases and increased absences [10]. Another feature associated with male office workers in Poland is smoking, a habit socially and culturally present in this type of job for years [11].

Both overweight and obesity are related to an increased all-cause mortality [12]. Regular monitoring of body mass in clinical practice and primary prevention of CVD may be useful in identifying people with an increased mortality risk [13]. The latest data suggest that cardiorespiratory fitness (CRF) not only plays an important role in decreasing the mortality risk but also constitutes a prognostic value and protective value related to improving longevity and decreasing the occurrence of CVD and other coexistent diseases (e.g., hypertension, diabetes, depression) [14,15,16]. It has been shown that CRF estimated by means of exercise tests significantly improves the classification of both the short- and long-term CVD-caused mortality when it is added to traditional risk factors [17]. However, due to practical reasons, CRF tests are not routinely performed by cardiologists or primary care physicians— such as general practitioners (GPs). Hence, non-exercise methods of CVD-related mortality risk assessment should be given more attention, being practical and useful tools which are more and more commonly used due to their cost effectiveness and availability [18,19,20]. From the review of the studies by Wang et al. (2019) it follows that in the equations of non-exercising models for estimating CRF the following variables are most commonly used: age (95%), physical activity status (PAS) (80%), BMI (41.7%), gender (38.3%), resting heart rate RHR (30%), body mass (28.3%), WC (26.7%), body height (21.7%), smoking cigarettes (16.7%), %Fat (13.3%) [21]. There is an increasing interest in the development of models estimating the probability of CVD occurrence. In such models, individual, 10-year risk is estimated by means of measuring typical CVD risk factors [22]. There are already several formulas for the assessment of risk based on physical activity—they are generally designed for patients already exhibiting cardiac symptoms and they combine grading based on physical activity with the results of additional tests [23]. There are also models which use objective assessment of exercise capacity to improve the precision of systematic coronary risk evaluation SCORE [24].

One of the models, the FIT Treadmill Score (“FIT” refers to Henry Ford ExercIse Testing (FIT) Project), deserves special mention due to its simplicity. Developed by a team of cardiologists from the Johns Hopkins University, it can be dedicated to early primary prevention. The FIT Treadmill Score formula analyses mortality risk based on age, sex, fitness level measured with the peak heart rate during exercise and the MET values obtained during that exercise [25].

Our studies, similarly to others [26,27], support initiatives aiming at the improvement of the general health condition in the workplace and may turn out to be helpful in identifying modifiable risk factors for future cardiovascular events among working males.

The aim of the study was to develop a prediction model for FIT Treadmill Score that only depends on easily accessible somatic data and smoking without the need to perform the exercise test anymore. The FIT Treadmill Score scale is calculated by means of the exercise test result, which may be inconvenient in clinical and preventive practice. The simplicity of the FIT Treadmill Score formula lies in classifying the tested people into four CVD risk groups, based on a clear point system. In our opinion, this way of risk assessment is well suited for primary prevention, however, it requires the performance of an exercise test. The problem here may consist in the availability of the treadmill or the cycle ergometer with a MET calculator as well as in the time needed for the performance of the test in healthcare facilities, which limits the scope and the possibility of performing and using it commonly in primary prevention.

The idea to create a simple CVD risk calculator for symptomless, working-age men, based indirectly on the FIT Treadmill Score scale resulted from the fact that a non-exercising model of CVD risk assessment based on such a point formula as the one suggested by a team of cardiologists from the Johns Hopkins University was not present in the available literature. The computations of the FIT Treadmill Score analogue by means of easily available indices seem, in our opinion, to bridge this gap, making this scale more accessible in everyday practice for the quick and economical diagnosing of CVD risk.

## 2. Materials and Methods

The study covered 146 working men aged 26–60, recruited from the biggest workplaces in south-eastern Poland during periodical check-ups, who consented to the participation in the study. The inclusion criteria consisted a negative cardiological history as obtained by a qualified occupational medicine physician as well as having a desk job. While the medical history was being taken, information was also obtained on smoking cigarettes and on the character of the work done. The exclusion criterion consisted in the affirmative answer to any of the questions from the Physical Activity Readiness Questionnaire (PAR-Q). The studies were performed in the Medical College of Rzeszów University, in the Diagnostic Laboratory for Sports and Health Training. Prior to the exercise test, selected anthropometric measurements were taken. Body mass and its components were determined by means of the bioelectrical impedance analysis (BIA) with the use of the Tanita TBF 300 electronic scale, body height was measured with a stadiometer (SECA 213, Hamburg, Germany) with accuracy to 1 mm and waist circumference with a constant tension tape measure compliant with the WHO protocol [28]. WHO’s STEPwise Approach to Surveillance recommends the measurements be taken between the lower margin of the last palpable rib and the top of the iliac crest.

For the analysis, the obesity measures most commonly used in epidemiological studies were applied: body mass index (BMI), waist circumference (WC) waist to height ratio (WHtR) and body mass fat (Fat%) [29,30].

The exercise test was performed according to a 50 W/2′/25 W graded protocol with the use of Kettler DX1 Pro cycle ergometer connected to a computer set joined with a 12-lead EKG MEDEALINE 3. The starting workload of 50 watts (W) was gradually increased by 25 W every 2 min. The task of the person being tested was to maintain the revolutions within the range of 60–70 per minute (rpm). The criterion for completing the requirements of the test was volitional exhaustion and the inability to maintain the working speed within the range of 60–70 rpm. The participants were encouraged to achieve the maximum target heart rate appropriate for their age (according to the formula: 220-age). Maximal heart rate (HRmax) was defined as the peak heart rate achieved at the end of the exercise. The peak metabolic equivalents of task (METs) were determined on the basis of the highest workload and the body mass of the tested person, with 1 MET being the equivalent of 3.5 mL of oxygen uptake/kg/min.

For calculating the mortality risk for men, an algorithm developed by a team from Johns Hopkins Ciccarone Center for the Prevention of Heart Disease was used, in the following formula:

**FIT Treadmill Score = %MPHR + 12 × MET − 4 × Age [years]**

%MPHR = percentage of maximum predicted HR

The measure values are usually within the range (−200; +200) and the lower values indicate a higher mortality risk. The values within the range (100; 200) indicate that a given person has a 98% estimated likelihood of surviving the next 10 years, while the value within the range (−200; −100) indicates that a given person has a 62% chance of survival. The following classification is adopted (Table 1.) [25].

### Statistical Analysis

The statistical analysis consisted first of all in the description of the distribution of the somatic features and the FIT Treadmill Score measure in the study population by means of descriptive statistics. The distribution of the FIT Treadmill Score was presented in graphic form, with categorisation into four intervals with a specified mortality risk. The correlation between the FIT Treadmill Score and the age was presented as well. With the use of linear regression analysis, a model was created, allowing for the estimation of the FIT Treadmill Score on the basis of age, body build and the fact of smoking cigarettes. The optimal shape of the model was searched for by means of stepwise regression, selecting the variables from among their wider selection—most of all other indices of body build. Next, a group of people with the 11% mortality risk was isolated and factors determining belonging to that group were searched for by means of a logistic regression model. The estimations of the parameters of the regression models were provided with the 95% confidence interval (applying also to the mean values for the measures analysed). Results with the *p* value below 0.05 were considered statistically significant.

The computations and the diagrams were made by means of STATISTICA v. 13 (StatSoft, Tulsa, OK, USA).

The study was performed in compliance with ethical standards as specified by the Declaration of Helsinki. The study had also obtained the consent of the Bioethics Committee of the University of Rzeszów—Resolution no. 8 May 2019.

## 3. Results

### 3.1. Characteristics of the Study Population

The analysis concerns 146 men aged 26–60. The basic information about the study group—in particular the information about the distribution of somatic features and indices—has been presented by means of descriptive statistics (Table 2).

The mean BMI amounted to approx. 26.4, which indicates quite common occurrence of overweight (affecting at least half of the men). WHO criteria were used as a reference for BMI values [31].

Among the study population there were 25 smokers, which constituted approx. 17% of all the tested men.

### 3.2. Mortality Risk Measure and Its Classification

**Figure 1:**
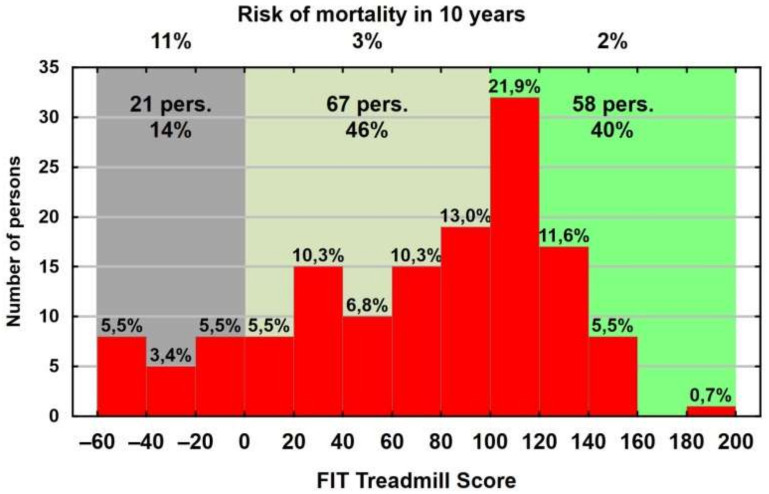
Distribution of FIT Treadmill Score and risk of mortality classification in 10 years.

The lower measure for FIT Treadmill Score values refers to higher mortality risk. Table 3 and the diagram (Figure 1) present the information on the distribution of the death risk measure and its classification into four categories suggested by its authors [25].

The mean value of the FIT Treadmill Score in the study group amounted to 69.4, with the median of 82.6. As the median is larger than the mean, we might speak about the negative skewness of the FIT Treadmill Score distribution, which is also well visible in the histogram. The diagram also provides the classification of the FIT Treadmill Score with respect to the 10-year mortality risk—as seen, there are no persons with the death risk at the level of 38%, and approximately every seventh man has the 10-year mortality risk at the 11% level (21 persons, i.e., 14%). The 10-year mortality risk at the level of 3% occurred in 46% (67 persons), and at the level of 2%, in 40% of the men (58 persons). The mean age of the men in those groups amounted to 30.3, 41.0 and 54.9 years, respectively.

**Figure 2:**
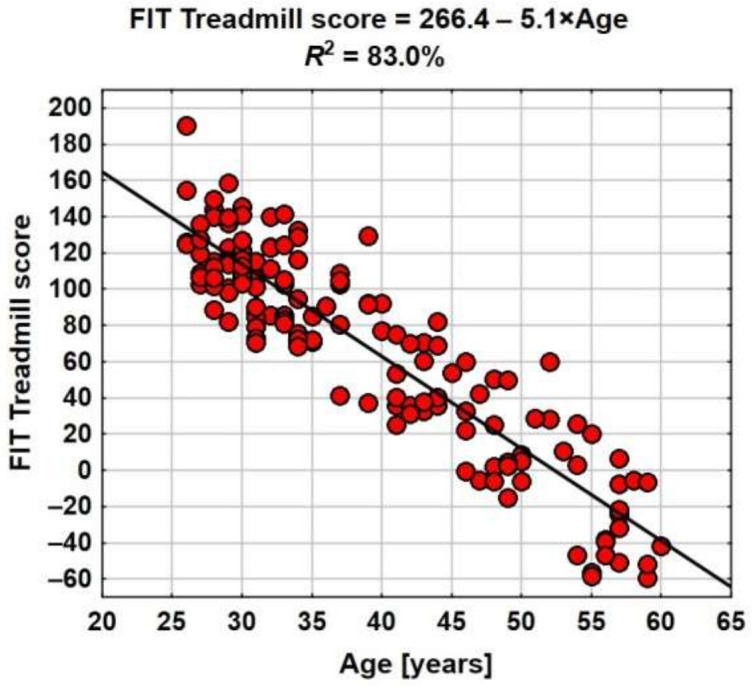
Distribution of FIT Treadmill Score and risk of mortality classification in 10 years.

The scatter graph shows the correlation between the mortality risk measure and the age. From the regression model (Figure 2) it follows that with each year of age, on average, the FIT Treadmill Score value drops by 5.1 points.

In the formula by means of which the FIT Treadmill Score is computed, the coefficient −4 appears next to age, and in the estimated regression equation the coefficient is −5.1.

Age is a factor significantly affecting the FIT Treadmill Score, hence all further analyses shall be conducted taking it into consideration.

### 3.3. Factors Related to the Lifestyle vs. Point Measure of Mortality Risk

In order to estimate the FIT Treadmill Score merely on the basis of somatic data and the medical history, it was tested by means of regression analysis to what degree lifestyle-related factors—indices related to obesity and the fact of smoking cigarettes—allow for estimating the measure of mortality risk. As independent features, the following were introduced into the linear regression model: age, BMI, Fat%, waist circumference and the WHtR as well as a dichotomic variable related to smoking cigarettes.

By means of the forward stepwise regression procedure a model was searched for which would account for the variability of the mortality risk measure in a given population, while including only statistically significant factors. The results obtained were presented in the form of a formula allowing for the prediction of the FIT Treadmill Score as well as in the tabular form, containing more detailed information on the results of the regression analysis.

The model which describes the variability of the mortality risk best contains information about age, smoking cigarettes and the WHtR (Table 4).

This model allows us to account for a significant, i.e., over 87%, part of the mortality risk variability measure in the tested population. Based on the value of the standardised regression coefficient *β* it could be stated that age is the factor that determines the mortality risk measure to the largest extent, followed by the WHtR and smoking cigarettes.

Estimating the non-exercise model of the FIT Treadmill Score value for a given male person is possible on the basis of the following formula:

FIT Treadmill Score = 390.95 − 4.58 × Age [years] − 11.20 × Smoking cigarettes − 2.64 × WHtR

It should be remembered that the value thus calculated is only a prediction of the value determined on the basis of the exercise test, with standard error of the estimation amounting to ±19.1 points.

### 3.4. Somatic Factors vs. Mortality Risk Classification

The creators of the FIT Treadmill Score measure suggested the division into four categories for which the likelihood of cardiovascular-related death was estimated (see Table 1). In our studies, in the tested population 21 men (approx. 14%) were classified into the 11% mortality risk group. Using the same independent factors as in p.3.3, a model determining belonging to that group was searched for. To that end a logistic regression model was applied and by means of the forward stepwise regression procedure the optimal model was searched for. An estimation of the FIT Treadmill Score point value based on the regression model suggested in the previous point does seem a better approach, since the value of 5 points has a different weight than 95 points, though according to the classification they both mean the same 3% mortality risk. Nevertheless, it is worth suggesting a tool which would allow for a direct estimation of the likelihood of belonging to the group with the highest, 11% mortality risk without estimating the FIT Treadmill Score point measure.

Below are the results of the logistic regression in the tabular form (Table 5.) as well as in the shape of the formula that allows for determining the likelihood of the 11% mortality risk for any male. The model contains the same features as in the linear regression model. The predictive power of the model is good (AUC as high as 0.986).

The likelihood of being classified into the group with the 11% mortality risk may be calculated according to the following formula, which could be easily implemented, e.g., in a spreadsheet (in this formula smoking cigarettes is a dichotomous variable: 1—smoking, 0—no smoking).
*p* =exp(−42.8530 + 0.5051 × Age [years] + 4.5376 × Smoking cigarettes + 0.2720 × WHtR)1 + exp(−42.8530 + 0.5051 × Age [years] + 4.5376 × Smoking cigarettes + 0.2720 × WHtR)

If there are no grounds for accepting different rules, a given person is classified into the 11% mortality risk group if P computed from the formula below exceeds 0.50. The measure of the goodness of fit of the model to the data in logistic regression consists in the classification table (Table 6.). As it can be noted, by means of the logistic regression model being discussed, 142 out of 146 persons were correctly classified, which amounts to over 97% of accurate results.

## 4. Discussion

One of the most basic methods employed in the assessment of cardiorespiratory fitness is an exercise test performed with the use of a treadmill or a cycle ergometer [32,33,34].

In our study, to assess the reaction of the cardiovascular system to exercise a cycle ergometer was used due to the ease and safety of its use for less physically fit people and the results obtained were inserted into the formula suggested by Ahmed et al. (2015). It was assumed that the basic criterion for the completion of the exercise test was volitional exhaustion and the inability to maintain the number of revolutions on the cycle ergometer above 60 per minute. During the exercise test, in order to achieve the full engagement, the tested men were encouraged to achieve the maximum heart rate according to the formula 220-age—the formula which is commonly applied in exercise physiology and clinical practice [20,35]. This formula has its limitations, though, and it might not predict the target HRmax of a given person precisely [35], hence the criteria for completing the test were as follows: the refusal to continue the exercise and the inability to maintain the required number of revolutions per minute on the cycle ergometer. In the work by Ahmed et al. (2015) for the calculation of MPHR the formula 220-age was used and the median %MPHR achieved was 91%.

For an average person of working age with a negative cardiological history no cases with a 38% mortality risk were noted. That is why it seems that from the point of view of primary prevention for healthy males of working age, with desk jobs and a negative cardiological history, the key thing is to determine whether they belong to the group with the 11% risk of death within 10 years. Hence the plan to work out the non-exercise model. The accuracy of classification into the 11% mortality risk group based on the logistic regression model amounted to 97%.

Our study tested to what a degree lifestyle-related factors help to determine the mortality risk measure level. For that purpose, BMI, Fat%, WC, the WHtR, smoking cigarettes and age were introduced into the linear regression model as independent factors.

The factors considered by us in the model belong to the group of primary risk factors for cardiovascular diseases [36]. In the model describing best the variability of death risk there are age, smoking cigarettes and the WHtR.

A man who is 1 year older has his FIT Treadmill Score values lower by approx. 4.6 points. Smoking cigarettes translates, on average, to the decrease of the FIT Treadmill Score by 11.2 points, while the increase of the WHtR by 1 means a decrease of the modelled variable by 2.7 points. These factors may accumulate, hence, e.g., a person who is 5 years older, with their WHtR higher by 10 points and smoking cigarettes has their FIT Treadmill Score value lower by approx. 5 × 4.6 + 11.2 + 2.6 × 10 in comparison to a younger, slimmer and non-smoking person.

The presence of the WHtR in the regression model may be related to more and more reports on the importance of this ratio for complex cardiovascular health, called “early health risk” index [37]. The WHtR is indicated as a better predictor of cardiovascular disease risk than BMI and WC [30,38,39,40], however, contrary to them, it is not indicated as a common variable in equations for CRF predictive models [21]. Smoking is a well-documented factor increasing the risk of cardiovascular diseases [36,41]. It is also associated with the decrease in cardio-respiratory fitness [42]. Similarly, performing a desk job is often related to the decrease in cardio-respiratory fitness [43] and to the increase of cardiovascular risk [44]. Studies suggest that all the interventions increasing the level of CRF, including leisure time physical activity, increase the chance of reducing the cardiovascular risk for desk-job workers [45]. In our study, we did not consider the physical activity status (PAS). According to Wang et al. (2019), PAS is a variable which very often appears in CRF- estimating models. Recent studies also show that it is possible to create CRF-predictive models without considering PAS [20]. Prophylactic exams of the employees constitute a unique opportunity to conduct actions from the scope of health promotion as well as prevention in the case of healthy/symptom-free people. Unfortunately, occupational medicine physicians in Poland do not take enough advantage of these opportunities [46]. This simple model may provide them with help in this respect.

In the logistic regression model by means of which the 11% probability of mortality risk was modelled, there were the same factors as in the linear regression model. The risk of belonging to the 11% mortality risk group increases with age (1 year—OR = 1.66), with smoking cigarettes (OR = 93.47—this value is very high, but it is more cautious to use the lower estimation of the confidence interval being 3.82) and a higher WHtR (1 point—OR = 1.31). In the case of assessing the influence of several factors simultaneously, the OR values should be multiplied.

**Figure 3:**
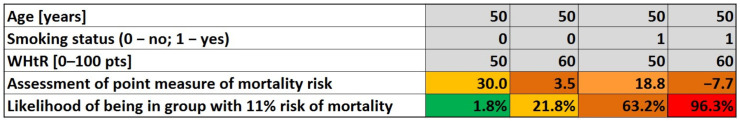
Assessment of point measure of mortality risk and likelihood of being in group with 11% risk of mortality in Excel for four hypothetical men—spreadsheet screen. WHtR—Waist to Height Ratio.

Based on the mathematical formulas obtained from both models it is possible to create a risk “calculator” for CVD occurrence, which may be useful for health monitoring—it does not require any complicated measurements, as the WHtR can be easily calculated on one’s own. It should be even less of a problem in a doctor’s office, without extending the duration of the doctor’s appointment significantly. The formula based on the linear regression model is extremely simple in terms of calculations, while the slightly more complicated formula from logistic regression may be easily implemented e.g., in Excel and used again and again (Figure 3).

Illustrating the risk in the way we suggested may become an incentive for people to increase their physical activity and improve their cardiovascular fitness. In the times of rising medical costs, it is worth promoting a tool which might motivate people to change their behaviour, at the same time being easy to use, particularly by GPs and occupational medicine physicians. The presented model, just like others relying on the prognostic value of the anthropometric measurements with reference to major adverse cardiovascular events [47], may complement primary prevention when laboratory tests are unavailable or difficult to perform.

### Limitations of Our Studies

A possible limitation of our study’s results lies in the fact that the exercise was performed with the use of a cycle ergometer with reference to the FIT Treadmill Score protocol [25]. The cycle ergometer as well as the treadmill are used equally frequently in exercise tests. However, the peak oxygen uptake during the exercise with the use of the cycle ergometer may be 5–10% lower in comparison with that achieved on the treadmill, due to the smaller mass of the muscles involved [33]. Nevertheless, the median of MET in our studies amounted to 10.8 and was similar to that (10 MET) from the studies of Ahmed et al. (2015). The original protocol of the exercise test employed for the calculation of the FIT Treadmill Score had already been modified in other studies [48]. The study was performed with a small number of participants, with no external validation cohort. There is a need for further studies based on a bigger and more varied group which would confirm or question our results. Limitation of this study consists in a lack of data on the participants’ physical activity, both work- and leisure-related. Another essential limitation of the presented models is the fact that they did not refer directly to cardiovascular-related deaths but to the estimation of the probability of their occurrence based on the FIT Treadmill Score. Hence, replacing the FIT Treadmill Score with the regression model suggested in our work creates an additional risk of overestimating or underestimating the probability of the occurrence of negative, cardiovascular-related health events.

However, the regression model was well adjusted to the data (the coefficient of determination amounted to nearly 89%, and the standard error of the FIT Treadmill Score estimation was 19.1 points).

## 5. Conclusions

The work presents a regression model allowing for the estimation of the point value of the FIT Treadmill Score established on the basis of the exercise-test, without the necessity of performing such a test. Besides, it suggests a new, simple calculator for predicting the occurrence of 11% mortality risk among apparently healthy men, based on the information about their age, smoking and the Waist to Height Ratio (WHtR). The variable WHtR included in the final equation turned out to be more predictive than the WC, BMI and Fat%, which constitutes a different perspective on the somatic variables considered so far in the applied algorithms of non-exercise models.

The simplicity of the model as well as resulting from it a possibility of its widespread use should allow for better health monitoring of working-age men, especially those with a desk job, in order to predict cardiovascular risk and, related to it, mortality risk, thereby improving the quality of public health management. A further study within the suggested model should consider the limitations mentioned in our work.

## Figures and Tables

**Table 1:** Risk of mortality in 10 years—classification based on FIT Treadmill Score.

FIT Treadmill Score	Risk of Mortality in 10 Years
≥100	2%
[0; 100)	3%
[−100; 0)	11%
<−100	38%

**Table 2:** General characteristics of the study population.

Age and Somatic Characteristics	Mean (95% CI)	Std. Dev.	Minimum	Median	Maximum
Age (years)	38.7 (37.1; 40.4)	10.0	25	30	60
BMI (kg/m^2^)	26.4 (25.9; 26.9)	3.3	16.9	26.1	34.7
Fat (%)	21.0 (20.2; 21.9)	5.3	3.8	20.9	39.1
WC (cm)	95.2 (93.7; 96.6)	8.7	70	96	117
WHtR	53.8 (53.0; 54.6)	4.9	41.2	53.7	67.5
Body height (cm)	177.0 (176.1; 177.9)	5.7	166	176	194
MET	10.7 (10.4; 11.0)	2.0	6.7	10.8	16.2
HR max (bpm)	176.4 (173.9; 178.9)	15.5	126	177	210

BMI—Body Mass Index; Fat—body mass fat; WC—Waist Circumference; WHtR—Waist to Height Ratio; MET—Metabolic Equivalent of Task; HRmax—maximal Heart Rate; 95% CI—95% confidence interval; Std. dev.—standard deviation.

**Table 3:** Descriptive statistics for FIT Treadmill Score.

	Mean (95% CI)	Std. Dev.	Min	Median	Max
FIT Treadmill Score	69.4 (60.3; 78.6)	56.1	−59.1	82.6	190.3

**Table 4:** Regression model for predicting FIT Treadmill Score.

Independent Features	FIT Treadmill Score		
	*R^2^* = 88.7%, *F* = 366.9, *p* ≤ 0.001, SSE = 19.1		
	B (95% CI)	*p*	*β*
Age (years)	−4.58 (−4.92; −4.24)	≤0.001	−0.82
Smoking cigarettes	−11.20 (−19.59; −2.81)	0.009	−0.08
WHtR (0–100)	−2.64 (−3.33; −1.94)	≤0.001	−0.23

*R*^2^—coefficient of determination, *F*—test statistic and *p* value for significance of whole model, SSE—standard error of estimation, *B*—regression coefficient with 95% CI, *ß*—standardise regression coefficient, *p* value.

**Table 5:** The logistic regression model for the occurrence of the 11% mortality risk according to the FIT Treadmill Score.

Independent Features	Modelling Probability of 11% Mortality Risk Occurrence AUC (95% CI): 0.986 (0.967–1.000)	
	OR (95% p.u.)	*p*
Age (years)	1.66 (1.26–2.17)	≤0.001
Smoking cigarettes	93.47 (3.82–2287.76)	0.005
WHtR	1.31 (1.02–1.69)	0.035

**Table 6:** Accuracy of the classification into the group with the 11% mortality risk based on the logistic regression model.

Prognosis of the 11% Mortality Risk Based on the Model	Observed Condition (11% Mortality Risk)	
	Yes	No
Yes	19	2
No	2	123
Total	21 (TPR = 90%)	125 (TNR = 98%)

TPR (sensitivity)—true positive rate, TNR (specificity)—true negative rate.

## Data Availability

The datasets used and/or analyzed during the current study are available from the corresponding author on reasonable request. A spreadsheet with relevant formulas may be shared on request.
